# Supplementary material for: The tardigrade damage suppressor protein binds to nucleosomes and protects DNA from hydroxyl radicals
Source: eLife. 2019 Oct 1;8:e47682. doi: 10.7554/eLife.47682 (PMC6773438; doi:10.7554/eLife.47682)
Supplement: Transparent reporting form [file elife-47682-transrepform.docx]

***eLife’s* transparent reporting form**

We encourage authors to provide detailed information *within their submission* to facilitate the interpretation and replication of experiments. Authors can upload supporting documentation to indicate the use of appropriate reporting guidelines for health-related research (see [EQUATOR Network](http://www.equator-network.org/%20)), life science research (see the [BioSharing Information Resource](https://biosharing.org/)), or the [ARRIVE guidelines](http://www.plosbiology.org/article/info:doi/10.1371/journal.pbio.1000412) for reporting work involving animal research. Where applicable, authors should refer to any relevant reporting standards documents in this form.

If you have any questions, please consult our Journal Policies and/or contact us: [editorial@elifesciences.org](mailto:editorial@elifesciences.org).

**Sample-size estimation**

- You should state whether an appropriate sample size was computed when the study was being designed
- You should state the statistical method of sample size computation and any required assumptions
- If no explicit power analysis was used, you should describe how you decided what sample (replicate) size (number) to use

Please outline where this information can be found within the submission (e.g., sections or figure legends), or explain why this information doesn’t apply to your submission:

N/A. This study did not involve the analysis of multiple biological samples.

**Replicates**

- You should report how often each experiment was performed
- You should include a definition of biological versus technical replication
- The data obtained should be provided and sufficient information should be provided to indicate the number of independent biological and/or technical replicates
- If you encountered any outliers, you should describe how these were handled
- Criteria for exclusion/inclusion of data should be clearly stated
- High-throughput sequence data should be uploaded before submission, with a private link for reviewers provided (these are available from both GEO and ArrayExpress)

Please outline where this information can be found within the submission (e.g., sections or figure legends), or explain why this information doesn’t apply to your submission:

Definitions of biological and technical replicates:

(taken from the NIH)

https://www.nigms.nih.gov/training/documents/module3-biological-and-technical-replicates.pdf

Biological replicates are parallel measurements of biologically distinct samples that capture random biological variation, which may itself be a subject of study or a source of noise.

Technical replicates are repeated measurements of the same sample that represent independent measures of the random noise associated with protocols or equipment.

In this study, each experiment was performed a minimum of two independent times with different preparations of Dsup. Because these experiments were performed with different preparations of Dsup that were derived from different biological samples (i.e., "biologically distinct samples"), we consider these experiments to be biological replicates.

In the paper, we have included this information in the Materials and methods at the end of the following subsections: "Gel mobility shift analyses with mononucleosomes"; "ACF-mediated assembly of periodic nucleosome arrays"; "Hydroxyl radical-mediated cleavage of nucleosomal DNA".

At the end of each section, it is stated: To ensure reproducibility of the data, each of the reported experiments was performed a minimum of two independent times with different preparations (*i.e.*, biologically distinct samples) of Dsup.

**Statistical reporting**

- Statistical analysis methods should be described and justified
- Raw data should be presented in figures whenever informative to do so (typically when N per group is less than 10)
- For each experiment, you should identify the statistical tests used, exact values of N, definitions of center, methods of multiple test correction, and dispersion and precision measures (e.g., mean, median, SD, SEM, confidence intervals; and, for the major substantive results, a measure of effect size (e.g., Pearson's r, Cohen's d)
- Report exact p-values wherever possible alongside the summary statistics and 95% confidence intervals. These should be reported for all key questions and not only when the p-value is less than 0.05.

Please outline where this information can be found within the submission (e.g., sections or figure legends), or explain why this information doesn’t apply to your submission:

N/A. This study did not involve the generation of data that requires statistical analysis.

(For large datasets, or papers with a very large number of statistical tests, you may upload a single table file with tests, Ns, etc., with reference to sections in the manuscript.)

**Group allocation**

- Indicate how samples were allocated into experimental groups (in the case of clinical studies, please specify allocation to treatment method); if randomization was used, please also state if restricted randomization was applied
- Indicate if masking was used during group allocation, data collection and/or data analysis

Please outline where this information can be found within the submission (e.g., sections or figure legends), or explain why this information doesn’t apply to your submission:

N/A. This study did not involve the allocation of samples into groups.

**Additional data files (“source data”)**

- We encourage you to upload relevant additional data files, such as numerical data that are represented as a graph in a figure, or as a summary table
- Where provided, these should be in the most useful format, and they can be uploaded as “Source data” files linked to a main figure or table
- Include model definition files including the full list of parameters used
- Include code used for data analysis (e.g., R, MatLab)
- Avoid stating that data files are “available upon request”

Please indicate the figures or tables for which source data files have been provided:

N/A. There are no additional data files.
